# Cofactors are Remnants of Life’s Origin and Early Evolution

**DOI:** 10.1007/s00239-020-09988-4

**Published:** 2021-02-06

**Authors:** Aaron D. Goldman, Betul Kacar

**Affiliations:** 1 Department of Biology, Oberlin College and Conservatory, Oberlin, OH 44074 USA; 2 Blue Marble Space Institute of Science, Seattle, WA 98154 USA; 3 Department of Molecular and Cellular Biology, University of Arizona, Tucson, AZ 85721 USA; 4 Lunar and Planetary Laboratory and Department of Astronomy, University of Arizona, Tucson, AZ 85721 USA; 5 Earth-Life Science Institute, Tokyo Institute of Technology, Meguro, Tokyo 152-8550 Japan

**Keywords:** Cofactors, Coenzymes, RNA world, Origin of life, Ancient life, Early evolution

## Abstract

The RNA World is one of the most widely accepted hypotheses explaining the origin of the genetic system used by all organisms today. It proposes that the tripartite system of DNA, RNA, and proteins was preceded by one consisting solely of RNA, which both stored genetic information and performed the molecular functions encoded by that genetic information. Current research into a potential RNA World revolves around the catalytic properties of RNA-based enzymes, or ribozymes. Well before the discovery of ribozymes, Harold White proposed that evidence for a precursor RNA world could be found within modern proteins in the form of coenzymes, the majority of which contain nucleobases or nucleoside moieties, such as Coenzyme A and S-adenosyl methionine, or are themselves nucleotides, such as ATP and NADH (a dinucleotide). These coenzymes, White suggested, had been the catalytic active sites of ancient ribozymes, which transitioned to their current forms after the surrounding ribozyme scaffolds had been replaced by protein apoenzymes during the evolution of translation. Since its proposal four decades ago, this groundbreaking hypothesis has garnered support from several different research disciplines and motivated similar hypotheses about other classes of cofactors, most notably iron-sulfur cluster cofactors as remnants of the geochemical setting of the origin of life. Evidence from prebiotic geochemistry, ribozyme biochemistry, and evolutionary biology, increasingly supports these hypotheses. Certain coenzymes and cofactors may bridge modern biology with the past and can thus provide insights into the elusive and poorly-recorded period of the origin and early evolution of life.

## The RNA World Takes Shape

All modern life shares the same fundamental genetic system, both biochemically and in terms of information processing. Genes are encoded on one type of molecule, DNA, transcribed into a similar type of molecule, RNA, but then converted to a completely unrelated molecule, proteins, through a vast translation machinery and by way of a genetic code. That all known organisms use a nearly identical genetic system (Crick 1958; Woese 1965; Crick 1968) strongly indicates that this genetic system evolved by the time of the last universal common ancestor (LUCA).

The origin and earliest evolution of life’s genetic system and its precursors remain an unsolved mystery in understanding the emergence of life on Earth. One compelling hypothesis is that this complex genetic system was preceded by a simpler information processing system that utilized RNA as its principal component (Visser 1984; Gilbert 1986). This so-called “RNA World hypothesis” was strongly supported by the discovery of RNA-based enzymes, or ribozymes, which demonstrated that RNA could serve a catalytic function in addition to its well-established informational function (Kruger et al. 1982; Guerrier-Takada et al. 1983). Even before the discovery of ribozymes, however, an early version of the RNA World hypothesis was being formulated (Woese 1967; Crick 1968; Orgel 1968). RNA is, after all, central to the modern genetic system, serving as a bridge between DNA and proteins. Its key role in the translation process indicated that the translation system may have arisen from an RNA World scenario and that, prior to the evolution of protein synthesis by translation, RNA may have had the capacity to form functional molecules.

It was in this context that Harold White (1976) would propose a second, metabolic line of evidence for an RNA World. White noticed that coenzymes, which play an essential role in metabolism, tend to be nucleotides or dinucleotides or are synthesized from nucleotides or nucleobases. He proposed that these coenzymes are relics of ancient ribozymes. Following the evolution of protein synthesis by translation, the surrounding structural scaffold of these ancient ribozymes was replaced by protein, leaving only the active site RNA behind as a coenzyme.

The discovery of ribozymes six years after White’s publication (Kruger et al. 1982; Guerrier-Takada et al. 1983) prompted the emergence of a new area of study for biochemistry centered on the identification and characterization of new natural ribozymes, as well as the engineering of artificial ribozymes capable of new functions (e.g., Bartel and Szostak 1993; Ekland and Bartel 1996; Unrau and Bartel 1998; Kumar and Yarus 2001; and many others). Compared to the discovery of ribozymes, the impact of White’s coenzyme hypothesis on origin of life research has been less conspicuous, but still very influential. The hypothesis has motivated various other thought-provoking hypotheses about the relationship between cofactors and early evolution and has been supported by a number of studies representing a range of disciplines, which we briefly summarize below.

## An RNA World within the Proteome

**Fig. 1:**
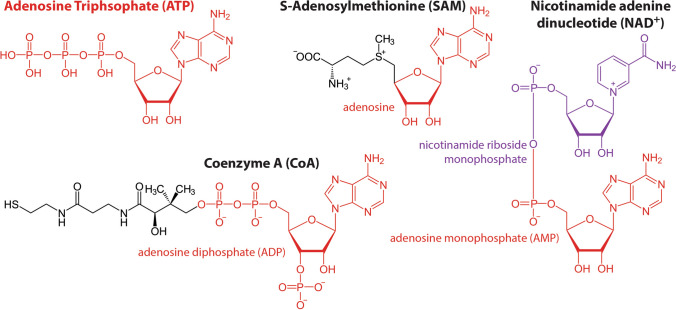
Several prominent group transfer cofactors composed of or derived from nucleotides. ATP is both a group transfer cofactor and the adenosine monomer added to RNA. NAD^+^ is a dinucleotide composed of adenosine monophoshate and the non-nucleic acid nucleotide, nicotinamide riboside monophosphate. CoA is composed of an adenosine diphosphate nucleotide attached to a cysteamine group (derived from cysteine amino acid) with pantothenate in between. SAM is composed of an adenosine monophosphate attached to a methionine amino acid. Chemical structures for this figure are based on black and white vector images available from wikimedia commons

While many protein enzymes operate without the requirement of a cofactor, others rely on non-protein cofactors for their catalytic functions. Cofactors may be inorganic, such as metal ions or iron sulfur clusters, or may be composed of organic or metallo-organic compounds. Organic cofactors are often referred to as coenzymes. Some of these coenzymes are further categorized as group transfer cofactors, which pick up a functional group as part of one reaction and release that functional group in a later, separate reaction. The most common group transfer cofactors across all three domains of life are either composed of nucleotides or derived from nucleotides (Fig. 1). Harold White’s key insight was to recognize this central role of nucleotide-derived cofactors across all domains of life and relate it to the nascent hypothesis that would be called the RNA World a decade later.

The quintessential group transfer cofactor, probably the first group transfer cofactor any biology student learns about, is adenosine triphosphate, or ATP. ATP is typically formed from the addition of a third phosphate onto adenosine diphosphate (ADP) through either substrate level phosphorylation or through the ancient ATP synthase motor complex (Gogarten and Taiz 1992). The third phosphate on ATP is unstable and its hydrolysis is also favored due to the intracellular disequilibrium achieved by high rates of ATP synthesis. Its transfer to another molecule can be used to drive energetically unfavorable metabolic reactions, to temporarily alter protein structures, or to facilitate other essential metabolic or physiological tasks. Notably, ATP is, itself, one of the four nucleotide monomers used for RNA synthesis.

Another prominent class of group transfer cofactors comprises the equally ancient and ubiquitous electron transfer compounds, such as NADH, NADPH, and FADH_2_, which are composed of dinucleotides, along with FMN, which is a mononucleotide. These cofactors are essential components of energy metabolism and the redox reactions that drive the synthesis of biomolecules. Two other group transfer cofactors that are central to metabolism are coenzyme A (CoA), which transfers acyl groups, and S-adenosylmethionine (SAM), which transfers methyl groups. Both of these cofactors are synthesized from an amino acid and a nucleotide (and, in the case of CoA, a third compound called pantothenate, also known as vitamin B_5_). Like the electron transfer cofactors, CoA and SAM also play essential roles in both energy metabolism and biosynthetic metabolism. These group transfer cofactors only represent a handful of examples, but they demonstrate the centrality of nucleotide-derived coenzymes in the core metabolism of all organisms across the tree of life.

White’s analysis was not limited to these group transfer cofactors. White also identified thiamin, a catalytic cofactor responsible for various decarboxylation reactions and condensation reactions between aldehydes, as potentially derived from ribozymes because it contains a pyrimidine moiety. White also proposed that the amino acid, histidine, represents a relic of the RNA world. Histidine, though an amino acid, is biosynthesized from ribose 5-phosphate and ATP (for review, see Alifano et al. 1996). As such, its side chain contains an imidazole ring that resembles that of a purine nucleotide. Histidine plays a prominent role in the acid–base chemistry of many enzymes and is by far the most common amino acid found in the active site of enzymes (Ribeiro et al. 2020).

In sum, nucleotide and nucleotide-derived group transfer cofactors are a foundational component of metabolism and the nucleotide-like amino acid, histidine, plays an outsized role in enzymatic catalysis. White argued that the central role of nucleotide-derived cofactors in modern metabolism suggests a prominent role for RNA in the early evolution of metabolism. In modern metabolism, however, there are also many other cofactors that are not composed of nucleotides. As we discuss below, it is possible that some of these non-nucleotide cofactors may also be relics of early evolutionary history or even prebiotic chemosynthesis and carbon fixation.

## A Cofactor Perspective on the Origin and Early Evolution of Life

Beyond providing an additional line of evidence for an RNA World, White’s hypothesis also serves as an example of how classes of cofactors may be used to better understand early evolutionary history. Szathmáry (1999) would later use the idea of amino acid cofactors with nucleotide handles to propose a solution to an important problem in the evolution of protein synthesis following the RNA World. Protein synthesis requires both a genetic code to translate RNA sequences to protein sequences and an arsenal of proteins and ribosomal machinery to implement the translation process, which Szathmary argued were more likely to have evolved at separate times than at once. A recent study demonstrated that, indeed, multiple molecular systems may not be able to evolve at the same time in rapidly evolving populations (Venkataram et al. 2020). Szathmary proposed that the genetic code evolved first as a way to attach amino acid cofactors via nucleotide handles to specific codons within a ribosome (Szathmáry 1999). In this way, ribozymes could take advantage of the more diverse set of chemical moieties found among amino acids and, in doing so, could have pre-adapted the late stage RNA World for the evolution of protein synthesis by translation.

Ancient cofactors, especially inorganic ones, may also reflect the geochemical environment in which life originated and/or first evolved. For example, hydrothermal vents are considered a potential setting for the origin of life because the chemical gradients surrounding them could have generally promoted prebiotic chemistry (Baross and Hoffman 1985). The iron-sulfur minerals found within some types of hydrothermal vents could have helped convert carbon dioxide into organic carbon (Wächtershäuser 1992; Yamaguchi et al. 2014; Roldan et al. 2015; Li et al. 2018; Hudson et al. 2020), and the porous nature of certain hydrothermal chimneys (which may have contained iron-sulfur minerals) could have helped concentrate compounds and served as an early form of compartmentalization prior to the evolution of phospholipid membranes (Martin and Russell 2003).

Many enzymes today, particularly redox enzymes, include inorganic iron-sulfur clusters which usually facilitate electron transfer but sometimes perform other functions in proteins (for a recent review, see Lill 2009). Martin and Russell (2003) propose that the evolution of iron-sulfur clusters and the membrane-associated proteins that use iron-sulfur clusters was a necessary step before life could unmoor itself from the geochemical setting of its origin. The implication of this hypothesis is that iron-sulfur cluster cofactors are a remnant of early evolution and their catalytic role in modern enzymes reflects the kind of chemistry that facilitated life’s origin in the first place (See Lane and Martin 2012; Sojo et al. 2016; but also Jackson 2016).

Various other organic and inorganic cofactors may have also played an important role in the origin of life. A variety of porphyrins, a class of organic cofactors that bind metals, which includes well known cofactors such as chlorophyll and heme, can be synthesized abiotically (e.g. Hodgson and Baker 1967; Lindsey et al. 2011) and may have been prominent during the early evolution of metabolism. The so-called Zinc-world hypothesis proposes that ZnS promoted prebiotic chemistry by facilitating light-driven CO_2_ reduction (Mulkidjanian 2009) and that the prevalence of zinc in ribozymes and ancient enzymes reflects this history (Mulkidjanian and Galperin 2009). Lastly, some studies support a prebiotic synthesis of pyridoxal phosphate (Austin and Waddell 1999; Aylward and Bofinger 2006), a very broadly used catalytic coenzyme, though an actual prebiotic synthesis has not yet been achieved.

**Fig. 2:**
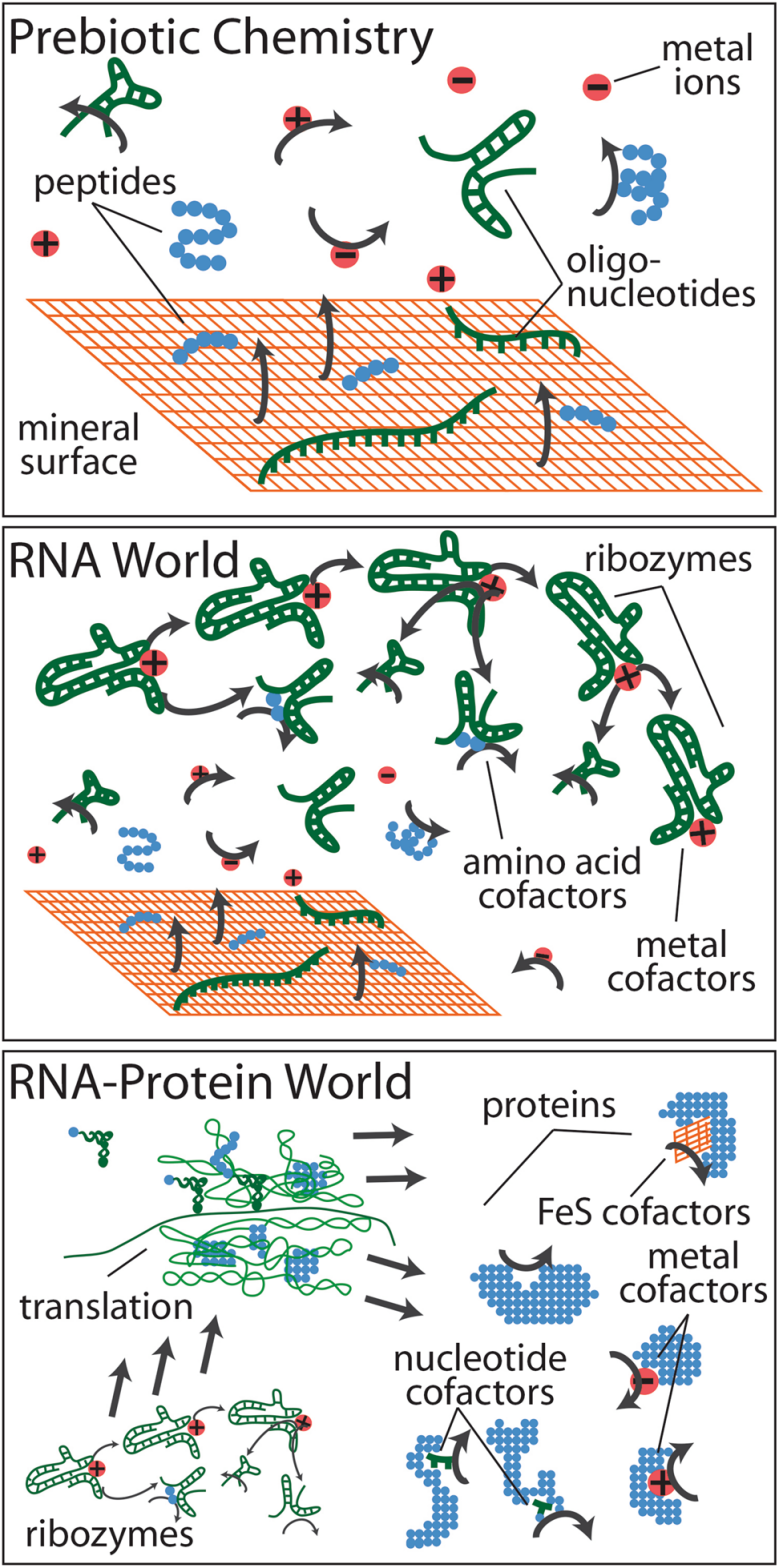
A general scheme for the emergence of different cofactors during the origin and early evolution of life. **a** A mineral surface (orange grid), for example FeS, and nearby ions (red circles), catalyze the formation of polymers such as peptides (blue) and oligonucleotides (green), which themselves may have catalytic properties. **b** An RNA world scenario emerges from within this geochemical context. **c** protein synthesis by translation evolved within the RNA-based genetic system and the resulting proteins retained cofactors that reflect the preceding RNA-based metabolism as well as the geochemical setting in which it first originated and evolved. This image is adapted from Goldman et al. (2016), published under open access in the Journal of Molecular Evolution

Taken together, these hypotheses portray a series of transitions from prebiotic geochemistry, to an evolving RNA-based genetic system, to the advent of protein synthesis by translation (Fig. 2). At each stage, the catalysts that facilitate these transitions are retained as cofactors, all of which are still central to protein-mediated metabolism, today. It is a compelling idea that fragments of the origin and early evolution of life are retained as catalytic and group transfer cofactors in the modern proteome. More recent research has begun to shed light on whether certain extant cofactors are, in fact, relics of early life.

## Supporting Evidence and Future Directions

The general hypothesis that certain cofactors reflect a historical role in the origin and early evolution of life can be tested through several independent and complementary avenues of research. Prebiotic chemistry can help to determine whether and in what geochemical environments certain coenzymes or enzymatic motifs may have been available as well as whether they were catalytic under prebiotic conditions outside of an active site of a protein enzyme. Some coenzymes, such as porphyrins (Hodgson and Baker 1967; Lindsey et al. 2011) and potentially pyridoxal phosphate (Austin and Waddell 1999; Aylward and Bofinger 2006), may have been prebiotically available, and certain nucleotides can be synthesized abiotically (Powner et al. 2009, 2010; Stairs et al. 2017; Yi et al. 2020). These compounds could have played a role in the origin of life if they were catalytically active outside of an enzyme’s active site.

White argued in his 1976 article that nucleotide-derived coenzymes may not be catalytic on their own because they evolved from ribozyme active sites and later adapted to enzyme active sites, and thus may require the context of a surrounding enzyme. This seems to be true of coenzyme A, which is stable under a range of probable early Earth conditions (Maltais et al. 2020) but does not appear to facilitate acyl group transfer. NADH, on the other hand, has recently been shown to transfer electrons to an FeS cluster within a simple peptide, after which, the electron can then be passed to ubiquinone (Bonfio et al. 2018), thereby abiotically mimicking the activity of Complex I in the respiratory electron transport chain.

These insights from prebiotic chemistry do not actually test White’s hypothesis, which was about the transition from an RNA world to protein-mediated metabolism. But research on ribozymes and riboswitches suggests a potential role for nucleotide-derived cofactors in an RNA world. Ribozymes, that are capable of self-incorporating coenzymes have been discovered (Breaker and Joyce 1995) and other ribozymes have been engineered to synthesize CoA, NAD, and FAD (Huang et al. 2000). In addition to this line of evidence from artificial ribozymes, some nucleotide derived cofactors such as NADH, FMN, SAM, and TPP are known to bind to naturally occurring riboswitches in vivo (Cochrane and Strobel 2008; Sherlock and Breaker 2020). Riboswitches are stretches of noncoding mRNA that moderate transcription or translation usually, but not always, through the binding of a ligand and a subsequent change in mRNA structure. Some have argued that riboswitches may have played a regulatory role in the RNA world (Breaker 2012). Furthermore, the coenzyme binding capabilities of riboswitches are thought to represent an ancient capacity for coenzyme-mediated catalysis in an RNA world metabolism (Cochrane and Strobel 2008).

In addition to these observations from prebiotic chemistry and ribozyme research, evolutionary analysis of ancient protein families suggests that early protein enzymes likely used cofactors in general, as recently demonstrated for zinc- and molybdenum-specific metalloenzymes (Kacar et al. 2017, Garcia et al. 2020), and nucleotide-derived cofactors, specifically (Goldman et al. 2013; Kirschning 2020). In one set of studies, Caetano-Anollés and colleagues chronologically ordered protein structures as defined by the SCOP database (Murzin et al. 1995) from most ancient to most recent (Wang et al. 2007; Caetano-Anollés et al. 2009). Among the ten most ancient of these protein structures (Goldman et al. 2010) are the P-loop containing hydrolase fold and the adenine nucleotide alpha hydrolase fold, both of, which are involved in ATP hydrolysis as well as other functions; the flavodoxin-like fold, which utilizes cofactors such as NADH, NADPH, and FADH_2_; the S-adenosyl-L-methionine-dependent methyltransferases fold, which utilizes SAM; the ferredoxin-like fold, which is the primary protein fold that utilizes iron sulfur clusters; and the TIM beta/alpha barrel fold which is found in proteins performing an extremely broad range of functions and using a similarly broad range of coenzymes and cofactors (Goldman et al. 2016).

In particular, the TIM barrel protein architecture has been proposed as an early bridge between the RNA World and protein mediated metabolism (Goldman et al. 2016). Many protein superfamilies that fold into the TIM barrel structure appear to be ancient (due to their very broad taxonomic distribution) and have evolved an extremely broad range of enzymatic functions. Unlike other protein superfamilies, the range of molecular functions found in individual TIM barrel superfamilies is strongly correlated with the incorporation of new cofactors within that superfamily (Goldman et al. 2016). During the transition from an RNA World to protein mediated metabolism, TIM barrel proteins may have provided a somewhat universal active site into which different ancient cofactors could be placed through evolutionary processes, thus transferring their catalytic properties from ribozymes (or minerals) into protein enzymes just as White proposed in 1976.

White’s hypothesis, as well as the broader hypothesis that cofactors in general reflect different stages of the origin and evolution of life, is supported by some laboratory studies as well as computational evolutionary analyses of extant protein families. However, the supporting evidence remains thin, most likely because these hypotheses have attracted less attention than other origin of life hypotheses over the last several decades. Even so, White’s hypothesis and the broader investigation of cofactors as relics of life’s origin and early evolution represent a conceptual foundation for integrating different disciplines across the study of early life.

Prebiotic chemistry is a powerful way to explore possible origin of life scenarios, but even a successful result will only amount to a proof of principle rather than a historical claim. Research on early evolution provides a form of historical evidence, but is currently unable to investigate evolutionary events that occurred during and following the emergence of the first life forms. Exploring protein cofactors as relics of life’s origin and early evolution is one potential way to bridge the divide between prebiotic chemistry and early evolutionary history. By bringing these two approaches together, it may be possible to combine their strengths and gather evidence about the origin of life as it most likely occurred given the subsequent evolutionary history. It is in this regard that White’s hypothesis is a powerful one because, as demonstrated above, it provides a unifying theme across otherwise disparate lines of research, from prebiotic chemistry to ribozyme and RNA biochemistry to evolutionary biology.
